# The temporal nature of social context: Insights from the daily lives of patients with HIV

**DOI:** 10.1371/journal.pone.0246534

**Published:** 2021-02-11

**Authors:** Gemmae M. Fix, Eileen M. Dryden, Jacqueline Boudreau, Nancy R. Kressin, Allen L. Gifford, Barbara G. Bokhour

**Affiliations:** 1 US Department of Veteran Affairs Center for Healthcare Organization and Implementation Research (CHOIR), Bedford and Boston, Massachusetts, United States of America; 2 Boston University School of Medicine, Boston, Massachusetts, United States of America; 3 University of Massachusetts School of Medicine, Worcester, Massachusetts, United States of America; Hunter College, UNITED STATES

## Abstract

**Background:**

Patients’ life contexts are increasingly recognized as important, as evidenced by growing attention to the Social Determinants of Health (SDoH). This attention may be particularly valuable for patients with complex needs, like those with HIV, who are more likely to experience age-related comorbidities, mental health or substance use issues. Understanding patient perceptions of their life context can advance SDoH approaches.

**Objectives:**

We sought to understand how aging patients with HIV think about their life context and explored if and how their reported context was documented in their electronic medical records (EMRs).

**Design:**

We combined life story interviews and EMR data to understand the health-related daily life experiences of patients with HIV. Patients over 50 were recruited from two US Department of Veterans Affairs HIV clinics. Narrative analysis was used to organize data by life events and health-related metrics.

**Key results:**

EMRs of 15 participants documented an average of 19 diagnoses and 10 medications but generally failed to include social contexts salient to patients. In interviews, HIV was discussed primarily in response to direct interviewer questions. Instead, participants raised past trauma, current social engagement, and concern about future health with varying salience. This led us to organize the narratives temporally according to past-, present-, or future-orientation. “Past-focused” narratives dwelled on unresolved experiences with social institutions like the school system, military or marriage. “Present-focused” narratives emphasized daily life challenges, like social isolation. “Future-focused” narratives were dominated by concerns that aging would limit activities.

**Conclusions:**

A temporally informed understanding of patients’ life circumstances that are the foundation of their individualized SDoH could better focus care plans by addressing contextual concerns salient to patients. Trust-building may be a critical first step in caring for past-focused patients. Present-focused patients may benefit from support groups. Future-focused patients may desire discussing long term care options.

## Introduction

Patients’ life contexts are increasingly recognized as germane to their health. The Social Determinants of Health (SDoH) framework—the “conditions in which people live and work”—provides a clinical approach to capturing patients’ life contexts [1, 2]. These contextual elements of peoples’ lives are known to affect health and unmet SDoH needs, and can impede patients’ abilities to adhere to recommended care [3, 4]. Health systems are increasingly screening for SDoH to identify contextual information about patients’ lives. Information about health-related social circumstances, like food insecurity or inadequate or unstable housing, is often collected using close-ended survey measures or Electronic Medical Record (EMR) reminders, where this information is then documented [2, 5, 6]. Patients with unmet needs are then ideally connected with resources [7].

Yet there are shortcomings to this clinical approach to capturing life context [7, 8]. These contextual elements were initially developed and largely identified by healthcare systems and researchers [1, 7, 9]. While important, a reliance on the patients’ current, health system-identified SDoH may be insufficient. Context, as noted by Duranti and Goodwin [10], is difficult to define and dependent on perspective. Garg et al. [7] remind us that topics like food insecurity, unemployment, and interpersonal violence can be sensitive; that patients should be engaged in if and how they want their needs met by the healthcare system; and further, once raised, patients need to be linked to resources that are typically beyond the scope of traditional clinical care. Initiatives to address SDoH may thus fall short if they are not aligned with patients’ perceptions and experiences.

Understanding patients’ life contexts may be especially important when patients have a multitude of complex medical and social needs, such as those associated with HIV. HIV treatments have improved, extending patients’ lives, but longer life expectancies mean that patients with HIV are increasingly developing age-related comorbidities like heart disease [11, 12]. Consequently, these comorbidities may be of greater importance to overall health than HIV status itself and are major provider concerns [13, 14]. Additionally, patients with HIV often have less education, higher rates of unemployment, and are more likely to live in poverty or experience homelessness [15]. The socio-cultural context of HIV may further present unique patient needs. HIV has historically been intertwined with blame, stigma, and marginalization. In the early days of the HIV epidemic, patients were blamed for their disease due to their sexual orientation or intravenous drug use [16]. The stigma persists; patients with HIV may thus be at particular risk of judgmental or stigmatizing communication with their providers [17, 18]. Conversations about patients’ needs should attend to their unique life contexts and histories.

Identifying which aspects of life context patients with HIV view as germane could help focus care planning and, considering the limited time of a clinical encounter, help providers know which resources are needed by which patients. Patient-identified contextual information can provide insights into the relationship between patients’ lives and health and presents an opportunity to explore how patients with a multitude of medical and social needs perceive, prioritize and self-manage their health. Further, EMR documentation of patient-identified contextual factors could facilitate patient-centered care across care teams responsible for managing comorbidities and social needs. In this study, we sought to first understand how aging patients with HIV describe their life contexts and how these relate to their health. Second, we explored if and how this context is documented in their EMR.

## Methods

### Overview

As part of a larger ethnographic study, we conducted both life story interviews [19] and collected EMR data to learn about life contexts and health of patients with HIV. Other aims of the ethnographic study examine provider experiences caring for patients with HIV, as well as if and how patient life contexts are discussed during clinical encounters. To assess these latter goals, we first sought to understand the life contexts of patients with HIV. All study procedures were approved by the Bedford VA Healthcare System Institutional Review Board.

### Data collection

Patients over age 50 with HIV and at least one comorbidity were recruited from two US Department of Veterans Affairs HIV clinics. We identified potential participants by reviewing the EMR for patients who were documented as receiving HIV care within the past year. Inclusion criteria included being over 50 and at least one comorbidity such as heart disease or diabetes. Opt-out cards were mailed to potential participants between January and November 2017. Twenty-three participants returned the opt-out cards, requesting not to be contacted by the research team. Those who did not respond were invited to participate.

We used life story interviews, a technique used in the social sciences, to gather information about participants’ life experiences, including their life history and current routines [19]. Our qualitative, life story interviews provided insights into participants’ lifeworlds and everyday experiences of their health [20, 21]. Although we were interested in their experiences with HIV and comorbidities, we started by asking about their life story, their daily lives in general and later focused on questions about their health and HIV. See Interview Guide. The semi-structured guide was used flexibly, with the interviewer following the interviewee’s lead; circling back later if topics were missed. The lead interviewer (GF) has doctoral training in anthropology, the disciplinary grounding of this study. At the conclusion of the interview, each participant completed a brief demographic survey which included race and sexual orientation. Interviews were conducted by phone or in-person, audio-recorded and professionally transcribed verbatim. Verbal informed consent was documented in a password protected digital file on the VA network.

Prior to the interview, the EMRs were used to identify eligible participants. After the interviews we reviewed the EMR for each enrolled participant. We recorded the 1) “active problem” and “active medication” lists and 2) HIV-specific information including HIV-RNA Detection for viral loads and CD4 counts. “Active problems” are part of the EMR coversheet and include diagnoses added by healthcare team members. These include conditions like coronary artery disease, hypertension, hyperlipidemia as well as mental health diagnoses like anxiety or depression. The problem list can include legacy conditions that were addressed but remain listed as “active,” as well as redundant or overlapping information like listing both “substance use” and “cocaine abuse.” We also reviewed “Progress Notes,” which provide appointment summaries, for dates ranging from one year prior to one year subsequent to our first interview.

### Analysis

Our analytic goal was to understand how patients conceptualized their life contexts to discern patterns and key contextual features germane to health. Because the interviews were semi-structured and prioritized following the interviewees’ lead, the content and flow varied. Therefore, we created case summary templates to systematically organize the interview and EMR data into similar formats. This facilitated comparison across participants. Summaries included key topics raised by the participants including trauma, past and current social activities, home life and descriptions of health priorities. EMR data about HIV status, active medications and comorbidities were collected for all cases and added to the summaries.

Next, we employed a narrative analytic strategy to identify narrative types depicting varying life contexts [22]. Unlike more traditional thematic analysis, this strategy does not disaggregate data and instead affords a holistic understanding of the interview data while retaining the patient voice [23]. Narrative analysis is a form of qualitative inquiry, often used in anthropology, that “juxtaposes disparate elements that belong together not by categorical similarity but because they contribute to the plot” [24]. This approach does not use traditional coding in which segments are identified across interviews for the same themes. Because our approach was more holistic and focused on each participant’s story, we used a template to systematically record data within each narrative. Three authors with training in anthropology (GF, ED) and public health (JB)) reviewed and then discussed data at weekly meetings. During one meeting, we noted that two participants had siblings murdered at a young age. One participant notably marked the event as something that happened in the past (“It was a long time ago, so if I sound cavalier about it, I’m not.”). Through further discussion about why there might be this nonchalant depiction of a significant event, we began to consider the narratives within a temporal framework. We then re-reviewed all 15 transcripts and accompanying analytic templates for further evidence of temporality. We then conducted a third review of the interviews to determine whether and how interviews fell along a temporal continuum. Discrepancies were discussed until consensus was reached.

Additionally, we explored if patient-identified contextual information was documented in the EMR. We conducted a fourth, further analysis of three exemplary cases, with a more in-depth EMR review including “progress notes.” These were examined for evidence of specific life-events and experiences described in the narratives.

## Results

The 15 participants were primarily male (N = 14), older (mean age, 63.7), and racially diverse (7 white; 6 Black, 2 Latinx). See Table 1 for a summary of all participants. Participants had, on average, 18.2 “active problems” and 10.2 “active medications.” Common comorbidities included a mental health condition (N = 14), cardiovascular disease (N = 10) and arthritis (N = 9). Six participants reported being gay in the post-interview demographics survey. We did not ask specifically about sexual orientation during the interview and participants did not always include sexual orientation as part of their narrative, nor did we observe recurring themes regarding identity-based stigma or discrimination. In the patient narratives, HIV was primarily discussed in response to interviewer questions rather than being raised by participants. A few participants clearly stated that HIV was not their priority. “Albert” (all participant names are pseudonyms), a 76-year-old white Male noted, “It’s almost like it doesn’t exist.” Participants brought up their comorbidities, often highlighting conditions that interfered with daily activities and sometimes contrasting them with HIV:

My body is starting to resist. And my knees, the fact that I’m having problems with my knees, that walking, you know, that even a simple thing like walking…takes a lot out of me. That is what’s hard. Not the HIV.

–“Brian”, 68-year-old white male

**Table 1:** Participant demographics & temporal orientation.

	Temporal Orientation						Total (N = 15)
	Past (N = 2)	Past-Present (N = 2)	Present (N = 4)	Present-Future (N = 3)	Future (N = 3)	Undetermined (N = 1)	
Male	2	2	4	2	3	1	14 (93%)
Mean age (years)	58.5	55.5	63.8	68.0	71.0	57.0	63.7
Race/Ethnicity							
White		1	1	2	2	1	7 (47%)
Black	2	1	1	1	1		6 (40%)
Latinx			2				2 (13%)
Sexual Orientation							
Heterosexual	2	1	3	2			8 (53%)
Gay			1	1	2	1	5 (33%)
Bisexual					1		1 (7%)
Not reported		1					1 (7%)
Social Context							
Marital status							
Married/partnered				1	1		3 (20%)
Single/Widowed	2	2	4	2	2	1	12 (80%)
Lives Alone	2	1	2	1	2	1	9 (60%)
Working				1	1		2 (13%)
Health status							
Avg. active diagnoses	18	16	24	12	11	11	19
Avg. medications	7	8	13	10	8	15	10
Detectable HIV			2		1		4 (27%)

### Temporal orientation

The participants had sometimes challenging social circumstances. Two-thirds lived alone; twelve were single. Only two worked; the remainder received government disability payments or were retired. Most importantly, however, we identified variation in the narratives according to the participants’ temporal focus in recounting their life story, with orientations to the past, present and future. Participants talked extensively about past trauma, current social engagement and concerns about future health. Despite the universality of these themes across the narratives, the weight participants assigned these experiences varied markedly. Narratives were thus organized by temporal focus (past, present and future). We then selected three exemplary narratives representative of these orientations for further analysis.

Below, we first describe each temporal orientation, followed by a deeper analysis of an exemplary case. Cases highlight how particular themes dominated the narratives within each orientation, as well as how events salient to participants were documented in the EMR. The three cases were all single men in their 60s who lived alone, and in addition to HIV had substantial comorbidities, with 11–24 “active problems” and 7–15 “active medications.” These superficially similar cases facilitated our exploration of how similar patients may differ in terms of orientation.

#### Past-focused

Past-focused narratives dwelled on unresolved experiences with social institutions such as the school system, military or marriage. These negative past experiences informed patients’ current experiences with social institutions like the healthcare system. Many described mistrusting their providers; their medical records documented poor adherence.

Carl was a 61-year-old Black, male. He described a history of negative experiences with social institutions from a young age, including with his family, the state Department of Child Services, the public-school system, the military and later, two marriages and the healthcare system. Carl noted that after his Mom’s death, despite having extended family, he was placed with the state:

My mom died when I was ten. She died in a car accident. And we were wards of the state [name]. And the state took us, strangely enough. But I did have relatives, and they say they came forward, but we ended up wards of the state.

His difficult childhood framed how he viewed his life. He connected his “really powerful and potent childhood” to a later traumatic military experience, in which a fellow service member committed a violent murder.

[The violence] can’t really be explained because [the other service member who committed the murder] was as calm, as, you know, like a pond in the morning. There wasn’t a ripple in him. There wasn’t a ripple in him. But it was that close to me, because I had been to his trailer… And that, that’s when I realized that in an instant, in a finger snap, anything, people have the potential to do anything.

These experiences reflect a core theme repeated throughout his narrative–that of mistrusting what you hear and see.

Carl’s past experiences with the untrustworthy child services system informed his current perspective on the healthcare system, which he viewed as part of a broad conspiracy. For example, during a description of his healthcare providers, he stated:

I know the same people that own the drug companies are the same people that own the hospitals, which are the same people who are running the same fourth branch of the government. Which, people are calling the shadow government. I know that this, I see the connectivity, and the control and the manipulation, you understand?

These concerns translated into mistrust of his providers’ recommendations for care, with a preference instead for deferring to God’s will. Carl ended his narrative by characterizing a time when his leg “swelled up as big as a telephone [pole]” and his providers raised the possibility of amputation. He stated that he refused their advice and went on to talk about God and nature taking its course. Similarly, when he discussed pain medications offered by his providers, he linked them to an ingredient in rat poison he attributed to being developed by the Nazis. He contrasted this with his preference for cannabis which is natural and put on earth by God.

This perspective was reflected in the EMR, where progress notes echoed the patient’s mistrust of providers and inconsistent adherence. In the example below, his mistrust of the system resulted in not believing the providers’ diagnosis of a skin problem and subsequently his non-adherence to his HIV medication. The initial note in the series says Carl discussed “developing a rash on his back about a week after last visit which he associates with receipt of the flu shot.” The doctor went on to write: “[The] rash is healing. Located directly under neck chains he is wearing and has appearance of contact dermatitis but [the patient] reports wearing these chains for a long time without problems.”

Two months later the provider wrote that the “rash on neck and extremities improved with (topical cream), requests refill.” While the patient appeared to have accepted a prescription for the cream, he seemed to resist the doctor’s diagnosis. Succeeding notes stated: “Pharmacy history showed he hadn’t refilled his HIV medication in several months… Patient retrospectively states he stopped medications because he thought it was making his skin more sensitive.”

Carl’s past-focused orientation engendered his mistrust of the pharmaceutical industry and healthcare system. These convictions facilitated an overall explanatory model of illness predicated on mistrust of the healthcare system and informed his belief that HIV medications caused his rash.

Similar mistrust was seen in other participants with past-focused orientations. Another, in describing the facility where he gets healthcare, stated his providers were financially motivated to harm patients:

There was a lot of nepotism going on. There was so much stuff going on up there, that they weren’t really practicing medicine… The doctor and the security guard and the nurse, they were killing off Veteran [patients] to collect insurance money.

56-year-old Black male

While another participant was deeply suspicious of an incident where the hospital changed his medication dosage without his being aware. He described mistakenly taking twice the recommended dosage and putting himself at risk of accidental overdose. He subsequently “got in a shouting match” with one of his providers.

#### Present-focused

In many of the present-focused narratives, participants described immediate barriers to everyday routines in their current lives. Their narratives documented loneliness, often compounded by declining mobility. For some, loneliness was exacerbated because they had stopped using substances–which previously had been a cornerstone of their social lives.

Daniel’s narrative exemplified this pattern; social isolation and loneliness permeated his interview. He was a 60-year-old white male with bipolar disorder, which he saw as the root cause of many of his issues, including his past alcoholism, HIV acquisition, and continued difficulties with medication adherence. He tied his drinking to lost family connections, never marrying or having children. He contrasted his present isolation with his life when he was drinking and manic, when he was the “center of attention” at the bar. Rather than just improving his health, sobriety resulted in diminished social relationships. He described attending Alcoholics Anonymous “to find people to associate with that didn’t drink, because everyone I grew up with and knew and was friends with, drank.” He lamented his current state of loneliness:

I wish I could pick up the phone and call so and so and say, ‘Let’s go down to [tourist area] and check out them little shops…’ But I pushed everyone so far out of my life, I didn’t even have anyone to be able to call to do that with… it was all my own doing, but I was lonely. I was. I was lonely. I’d go out and I’d take walks… by myself.

His other health conditions further exacerbated his social isolation. He had COPD which made it physically difficult to get around. Additionally, he was anxious his *Cardiac Defibrillator* might go off when he was out:

I have this defibrillator in my chest. So, I’m nervous at standing at the end of the platform for the train and crossing the street even, because this has gone off twice and saved my life twice. Once in a [convenience store] and once when I was just laying in my bed watching TV.

He worried leaving his house would endanger his health, but it also exacerbated his isolation.

Daniel’s EMR documented his bipolar disorder, anxiety and concerns about his defibrillator including how it “fired again” at the convenience store, but failed to mention the social isolation that permeated his narrative. While Daniel described loneliness and fear of going out due to medical conditions, the psychiatrist provided a medical diagnosis (agoraphobia) but did not mention the social isolation. The psychiatrist notes that while an anti-anxiety medication that had been prescribed for agoraphobia had been helpful, “he was still having problems going out in public and thinks that he is noticed by people.” This section ends with the psychiatrist stating that, “The patient has not been super reliable with his medications and he was encouraged to try to stick to the prescription.”

Daniel’s EMR demonstrated uneven healthcare use focused on his immediate concerns, such as those described above. His providers addressed these clinical concerns (mental health diagnosis and treatment), but did not address his social isolation and loneliness. Additionally, the patient’s EMR noted that he missed a number of longer-term prevention-focused appointments such as lung cancer screenings. This may reflect the patient’s de-prioritization of medical visits that were not directly relevant to his present circumstances, though they could impact future health. Neither his pattern of spotty engagement with the healthcare system, nor the implications for his future health, were noted in the EMR.

Other present-focused participants similarly described a connection between their substance use and fractured relationships. One 57-year-old white male, noted a loss of relationship with his son, while a 58-year-old Black male described the intersections of his own substance use, the overdose death of a spouse, strained relationships with his children and his currently living alone.

#### Future-focused

In the future-focused narratives, participants spoke at length about fears that future health challenges might limit engagement in meaningful aspects of their current lives. Despite features like being unmarried, childless or not having other traditional social networks, these participants reported being highly engaged in social activities like volunteering and hobbies that involved socializing. Yet they worried how further declines in their health might prevent future participation; notably, HIV was not one of those health concerns. Some were preoccupied with fears of death or dying alone. Several mentioned seeing therapists, in part, to deal with depression associated with aging.

“Ernest” was a 68-year-old Black male. His social life was a prominent part of his daily routines and narrative. Despite being “presently on retirement, social security,” he listed numerous part-time jobs. Additionally, on the day of the interview, among other activities, he had gone to a hospital-based HIV support group for gay men, participated in a singing club, and had dinner at a community center for people with HIV. He explained, “So I try to keep, I’d go crazy if I had nothing to do, so I try to keep active. I do a lot of social things.” HIV was rarely discussed; it was raised mostly in connection to HIV-related social groups. He noted that instead, aging was his primary concern:

I don’t even think about the virus… I never have no side effects, no problems with it. The problem of the virus doesn’t bother me. The problem is the things that do concern me are, I have issues with, is about aging, and aging and being alone.

Ernest was deeply concerned about what would happen to him as he aged and possibly being dependent on others. Reflecting on two women he mentored on service industry careers, he explained that they lacked common sense and worried that they typified the people who would care for him as he aged. He said:

‘Oh my God these are gonna be the people taking care of me in the nursing home.’ I worry, I worry, seriously I worry, I worry about aging. And because I have no relatives and, eventually, perhaps being in a nursing home.

He reflected on a time when he worked in a nursing home taking care of priests which required someone to clean up their urine and feces. He implied that if it was difficult to care for a priest, a gay man with HIV would likely be left soiled:

‘Aging-aging’ or ‘aging-*gay*-aging’? You know what I mean? Or just clearly aging period? Because here’s another thing too, being gay and aging, that’s another fear that we have going into a care facility with that and a lot of people’s ignorance, and with HIV.

In this example, his concern was not with the medical or biological consequences of HIV, but rather how persistent social stigma might affect his future treatment as an aging gay man with HIV.

He also worried about what might happen to him in a crisis alone in his home which would result in potentially losing mobility and thus his freedom and independence. He repeatedly gave detailed examples of scenarios that might happen that could end his current routines and permanently alter his future:

I have this rubber mat in front of my sink and if water gets underneath it, which happens, it becomes like a sheet of ice. You step on the top of it and underneath is wet and it just slides. And I see now one day I almost broke my neck. And I figured I could easily have broken, fell and broken something… And all of a sudden in a split second I’m in a wheelchair. …And then I wouldn’t be able to get into my apartment, you know what I mean?…And then all of a sudden my whole life is being rearranged because I have to find something. So it’s like, oh, the thought of getting old is just scary …

His concerns with aging and the possibility of future disability prompted him to seek healthcare. Ernest joked about his frequent utilization: “[My doctor] says, ‘You’re my healthiest patient and I see you more than my sickest.’ I’m like, ‘Listen, you told me I have a papercut, can I get a second opinion?’” This was corroborated by the comparatively large number of EMR progress notes demonstrating frequent appointments. However, aside from one social worker note about a support group in which Ernest discussed dying at length, Ernest’s preoccupation and worry about future needs or disability was not documented.

Patient life contexts, their perceptions and temporality may shift over time. Ernest’s current healthcare utilization contrasted his minimal use when first diagnosed with HIV.

When I first got diagnosed my T-cells was twelve hundred, eleven something, eleven hundred or something, okay, which is like almost healthy, like a healthy person, normal person. So I’m like, I’m outta here, catch me, I was like Roadrunner, beep, beep. So I didn’t go to the hospital, I had no need to go to the hospital. I just periodically went to check my T-cells. And after awhile it was still the same, so I was like, okay. I didn’t even think about it, you know what I mean. I was just waiting to get sick [from AIDS].

These descriptions hint at a shift in orientation as he aged. He described his youth and early diagnosis as a time that he was heavily using substances and thought he was going to die from AIDS. Once he realized he would survive, he started worrying about his future. This illustrates the possibility that temporal orientations shift across a patient’s life as s/he reconciles changing health and life contexts.

Similarly, other future-focused participants also talked about aging:

What’s limiting my life now is the fact that I’m getting older, and I don’t like that. I had a lot of friends who turned sixty before me. Not people with HIV necessarily, but people who turned sixty before me, and they all said something happens at sixty. I said, nah, I’m not gonna believe, I’m not buying this. But something has happened. I’m approaching sixty-nine now and I’m slowing down and I don’t like it.

68-year-old white male

## Conclusion

HIV is one of the most highly reported upon–and greatly stigmatized–diseases in recent history. Therefore it might be assumed that patients with HIV would be focused on their HIV status. Through our in-depth analysis of narratives of patients living with HIV, we found that HIV was only a small part of their daily lives. Others have found a variety of ways patients incorporate HIV into their identities [25, 26]. As Ho and Goh note, the salience of HIV may depend on their level of involvement with an “HIV community,” and comfort disclosing their HIV status to social networks. In our work, we found that participants were focused on different aspects of their life stories with differing temporal orientations toward past traumatic experiences, current social isolation and future consequences of aging. Our findings suggest these orientations informed how they thought about their daily life contexts and managed their health. Past-focused participants distrusted the healthcare system and demonstrated non-adherent health behaviors. In their narratives, we found repeated episodes of “structural violence,”–episodes of persons, because of their places within the larger social structure, disproportionally experience negative events [27]. These early experiences by our participants were still salient and informed ongoing interactions with the healthcare system. Those with adverse childhood experiences are less likely to trust medical professionals [28]. Previous work has found traumatic childhood events are common among both people living with HIV and Veterans [29–31] and identified a past-focused orientation in Veterans who have experienced trauma [32].

Those with present-focused orientations had immediate challenges in their daily lives like physical limitations and social isolation, the latter being increasingly recognized as deleterious to health [33–36]. We saw in our data that a present-focused orientation was related to a focus on immediate concerns, while ignoring forward-looking healthcare, like cancer screening.

Our future-focused case study example joked about his high healthcare utilization, yet his desire for long-term care planning was not addressed. Death and dying remain taboo topics [37, 38] while providers are incentivized to address immediate clinical tasks. Recognizing the difficulty of aging with HIV, Emlet et al. purposefully recruited patients over 50 who described themselves as “ageing successfully with HIV” [39]. In particular, they found that social support and community groups aided in resilience. Yet, for our participants, age-related declines in mobility were related to concerns that these supports might become difficult to engage with as they aged.

Our work has limitations. We lack information on how temporal orientations might change over time. Since we did not collect dates of diagnosis, we do not know the relationship between being diagnosed and temporal orientation. Others have found that self-identities changed in relation to time since diagnosis [40]. Further work should examine if and how orientation might change as well. In some of our interviews, participants’ temporal orientations were in transition. These orientations may therefore be fluid. Our study specifically focused on experiences of aging patients with HIV. Examining temporal orientations may be useful in younger patients living with HIV, or for other patients with complex, stigmatizing illness or chronic conditions.

Additionally, we compared interview narratives to clinical narratives documented in the EMR. This provided a fixed window into the patient-provider relationship and likely does not capture contextual information providers know but do not document. EMR documentation reflects about half of what actually happens during the clinical encounter [41]; thus clinicians may be aware of, and may also address patients’ life context issues, without documenting them. Yet, without this information in the EMR, some providers may miss key contextual information.

Healthcare systems are updating their EMR’s to better integrate patient information, including aspects of social determinants of health that are relevant to health, healthcare use, and adherence to recommended therapies. These features have the potential to humanize the patient, enhance the patient-provider relationship and build trust, by using features that emphasize the patient voice and goals [42]. The U.S. Veterans Health Administration–one of the world’s largest integrated systems–is in the process of changing to a new EMR and is actively working on strategies to incorporate patient context into the new system.

Our findings have lessons for clinical practice. Weiner notes the need to go beyond the medical model and assess patient context in a way that “considers the full breadth of the patient’s life circumstances” [43]. In our study, participants were similar (ie, single, aging men with HIV and comorbidities), but temporal orientations varied, suggesting patients would benefit from different kinds of conversations. Past-oriented participants were wary of the healthcare system, often non-adherent to recommendations and failed to keep appointments. Providers of past-oriented patients could focus on building trust through positive patient-provider relationships, listening to and discussing patient concerns. Present-oriented participants were focused on loneliness, and also missed appointments that required a forward-looking orientation to preventive health, such as lung cancer screening. Patients oriented toward current life may benefit from social support through social work programs or community resources. Our future-oriented participant joked about his high frequency of healthcare use, and his medical record confirmed this, yet his providers were not addressing his desire for long-term care planning. Future oriented patients like him may desire discussing how to stay active, long term care, nursing home options, or designating a power of attorney.

Identifying which contextual information is important to a particular patient can be challenging during a time-limited encounter with numerous clinical tasks. The preponderance of information about patient context and how it might inform health behaviors may feel distal and overwhelming. Instead of starting with the index condition of interest such as HIV, attending to patient narratives may facilitate more effective encounter. Listening for temporal variation in patient narratives may allow providers to tailor the encounter to patients’ unique needs and better focus care planning, based on what patients emphasize. Insight into patient contexts could be elicited simply by asking patients what matters most to them.

## Supporting information

S1 File (DOC)
