# Supplementary material for: The temporal nature of social context: Insights from the daily lives of patients with HIV
Source: PLoS One. 2021 Feb 11;16(2):e0246534. doi: 10.1371/journal.pone.0246534 (PMC7877603; doi:10.1371/journal.pone.0246534)
Supplement: S1 File — (DOC) [file pone.0246534.s001.doc]

**Interview Guide**

*This is a semi-structured, qualitative interview guide meant to be used flexibly. The interviewer may follow-up with further open-ended questions, requests for clarification or follow-up on issues raised by the participant. Questions may be asked in a different order, depending on the flow of conversation.* Sub-questions are prompts and may not be asked.

1. I’d like to start off the interview by learning a little bit about you and your history.
   1. Where do you live? Are you from this area?
   2. Can you tell me a little about your family?
   3. Do you live with anyone?
   4. Do you work?
2. Can you describe a typical day, for example what did you do yesterday?
3. Where does your health fit in?
   1. Can you tell me a little about your health in general?
   2. How has it changed over time?
   3. Are there aspects that concern you?
4. How does your HIV fit in to your overall health?
   1. Can you tell me a little about when you were diagnosed?
   2. What do you need to do to manage your HIV?
   3. Do you have other conditions?
   4. Can you walk me through your routines for taking care of your health?
   5. If you could have anything to help you better manage your health (other than a cure), what would it be?
5. Now, I’d like to ask a little about your healthcare.
   1. Who is the main provider you see?
   2. When you see your provider, what do you talk about? What kinds of problems does he/she help you with?
   3. Are there people in your life that help you, as well?
   4. Could you walk me through a typical appointment?
6. Is there something else that you thought I was going to ask today, but didn’t? Is there something else I should know?
7. If you had a magic wand and could change anything, what would it be?
